# Host factors do not influence the colonization or infection by fluconazole resistant *Candida *species in hospitalized patients

**DOI:** 10.1186/1477-5751-7-12

**Published:** 2008-12-16

**Authors:** Yun-Liang Yang, Ming-Fang Cheng, Ya-Wen Chang, Tzuu-Guang Young, Hsin Chi, Sai Cheong Lee, Bruno Man-Hon Cheung, Fan-Chen Tseng, Tun-Chieh Chen, Yu-Huai Ho, Zhi-Yuan Shi, Chung-Huang Hubert Chan, Ju-Yu Lin, Hsiu-Jung Lo

**Affiliations:** 1 Department of Biological Science and Technology, National Chiao Tung University, Hsinchu, Taiwan, Republic of China; 2 Department of Pediatrics, Veterans General Hospital-Kaohsiung, Kaohsiung, Taiwan, Republic of China; 3 National Yang-Ming University, Taipei, Taiwan, Republic of China; 4 Division of Clinical Research, National Health Research Institutes, Miaoli, Taiwan, Republic of China; 5 Section of Infection Diseases, Taipei Municipal Zen Ai Hospital, Taipei, Taiwan, Republic of China; 6 Section of Infection Diseases, Mackay Memorial Hospital Taitung Branch, Taitung, Taiwan, Republic of China; 7 Division of Infectious Diseases, Chang Gung Memorial Hospital, Keelung, Taiwan, Republic of China; 8 Section of Infection Diseases, Tainan Municipal Hospital, Tainan, Taiwan, Republic of China; 9 Section of Infection Diseases, Kaohsiung Medical College Chung-Ho Memorial Hospital, Kaohsiung, Taiwan, Republic of China; 10 Section of Infection Diseases, Buddhist, Tzu-Chi General Hospital, Hualien, Taiwan, Republic of China; 11 Section of Infection Diseases, Veterans General Hospital-Taichung, Taipei, Taiwan, Republic of China; 12 Department of Hematology and Oncology, Chang-Gung Memorial Hospital-Chiayi, Chiayi, Taiwan, Republic of China

## Abstract

Nosocomial yeast infections have significantly increased during the past two decades in industrialized countries, including Taiwan. This has been associated with the emergence of resistance to fluconazole and other antifungal drugs. The medical records of 88 patients, colonized or infected with *Candida *species, from nine of the 22 hospitals that provided clinical isolates to the Taiwan Surveillance of Antimicrobial Resistance of Yeasts (TSARY) program in 1999 were reviewed. A total of 35 patients contributed fluconazole resistant strains [minimum inhibitory concentrations (MICs) ≧ 64 mg/l], while the remaining 53 patients contributed susceptible ones (MICs ≦ 8 mg/l). Fluconazole resistance was more frequent among isolates of *Candida tropicalis *(46.5%) than either *C. albicans *(36.8%) or *C. glabrata *(30.8%). There was no significant difference in demographic characteristics or underlying diseases among patients contributing strains different in drug susceptibility.

## Background

Nosocomial infections caused by yeasts have increased significantly in the past two decades in Taiwan as well as other industrialized countries [1-4]. Infections by *Candida *species are important causes of morbidity and mortality in immunocompromised patients. The increase in the prevalence of fungal infections is closely associated with invasive medical procedures and the intense use of antibiotics in high-risk patients [5,6]. Resistance to antifungals has emerged in association with the increased use of these drugs [7-9].

The Taiwan Surveillance of Antimicrobial Resistance of Yeasts (TSARY) program was first implemented by the National Health Research Institutes (NHRI) in 1999 to prospectively assess the magnitude of the problem of yeast infection and drug resistance. Clinical isolates of yeasts were obtained from 22 geographically representative hospitals in Taiwan. Emergence of fluconazole resistant *Candida *species was documented in 1999 shortly after the surveillance program was instituted [9-11]. The survey revealed that 8.4% of the collected 632 clinical isolates were resistant [minimum inhibitory concentration (MIC) ≧ 64 mg/l] to fluconazole [12]. Furthermore, there is an association between the rate of fluconazole resistance and the number of non-albicans *Candida *species collected from different regions and hospital types [13,14]. This study was a follow-up, designed to determine whether intrinsic host factors of patients might influence the frequency of colonization or infection by fluconazole resistant strains.

## Methods

### Subjects

Yeast isolates were collected as described [15] from the 22 hospitals participating in TSARY in 1999. In principle, only one isolate was accepted from each patient. The number of clinical isolates with MICs ≧ 64 mg/l contributed from each hospital participating in TSARY program in 1999 ranging from none to ten [13,16]. Therefore, we chose hospitals contributed at least three clinical isolates with MICs ≧ 64 mg/l for this study. In total, there were 40 isolates with MICs ≧ 64 mg/l from nine chosen hospitals. However, medical information of five patients was not available. Thus, we have reviewed all 35 available ones. Patients with resistant strains were matched at a ratio of about 1:2 with those with susceptible strains according to the body sites from which the yeasts were isolated in the same hospital. Each hospital contributed from 7 to 21 patients into the study. Clinical data were recorded on standardized forms and analyzed according to demographic characteristics, hospital unit, duration of stay, predisposing factors, antimicrobial therapy, underlying illnesses, and laboratory findings. The definition of diabetes, hypertension, pulmonary diseases, bacterial infection, antifungal, antibiotics, antituberculous agents, dialysis, catheter insertion, and mobility affected were recorded when the *Candida *species were isolated. The outcome (mortality) was documented within three months after those stains were isolated. The protocol was approved by Institutional Review Board (IRB) of the National Health Research Institutes.

### Susceptibility tests

The MICs to fluconazole were determined according to the guidelines of the Clinical and Laboratory Standards Institute (CLSI) for *in vitro *antifungal susceptibility testing [12]. The RPMI medium 1640 (31800-022) provided by Gibco BRL was used for the testing. Strains from American Type Culture Collection including *Candida albicans *(ATCC 90028)*, Candida krusei *(ATCC 6258), and *Candida parapsilosis *(ATCC 22019) were used as the standard controls. The final growth of each isolate was measured by Biotrak II plate reader (Amershan Biosciences, Biochrom Ltd., Cambridge England) after incubated at 35°C for 48 hours. The MICs of fluconazole were defined as the minimum inhibitory concentrations of drugs capable of reducing the turbidity of cells to greater than 50%. Isolates with MIC ≧ 64 mg/l were considered to be resistant, whereas those with MIC ≦ 8 mg/l were susceptible. Isolates with MICs falling in between (16 – 32 mg/l) were susceptible-dose dependent.

### Statistical analysis

The chi-square test or Fisher's exact test were used for categorical variables. The Student T-test was used for continuous variables. Logistic regression was used to assess the independent effect of factors that were significant in the univariate analysis or were important to the association of interest. A probability (*P*) < 0.05 was considered significant.

## Results

A total of 35 patients from nine hospitals were selected and each one of them has contributed one clinical fluconazole resistant isolate. As a comparison, we have also reviewed 53 patients contributing susceptible isolates (with MICs ≦ 8 mg/l) from the same nine hospitals. Of the 88 isolates, there were three *Candida *species and their distribution in relation to the susceptibility to fluconazole is shown in Table 1. It shows that fluconazole resistance was found to be more frequent in *C. tropicalis *than either *C. albicans *or *C. glabrata*. The distribution of body sites from which the yeasts were isolated is shown in Table 2. Most isolates were from urine (52.3%) and sputum (23.9%) and candidemia was diagnosed in six patients (6.8%). Both *C. glabrata *and *C. tropicalis *were isolated more frequently from the urine than *C. albicans *(61.5% vs. 60.5% vs. 21.0%, respectively), whereas *C. albicans *was dominant in sputum than *C. glabrata *or *C. tropicalis *(47.4% vs. 15.4% vs. 18.6%, respectively).

**Table 1:** Distribution of fluconazole susceptibilities among *Candida *species isolated from patients in the nine hospitals

	***C. albicans***		***C. glabrata***		***C. tropicalis***		**Total**	
					
**MIC (mg/l)**	**Number**	**%**	**Number**	**%**	**Number**	**%**	**Number**	**%**
≧ 64	7	36.8	8	30.8	20	46.5	35	39.8
≦ 8	12	63.2	18	69.2	23	53.5	53	60.2
**Total**	**19**	**100**	**26**	**100**	**43**	**100**	**88**	**100**

MIC, minimum inhibitory concentration.

**Table 2:** Distribution of body sites of *Candida *species with different fluconazole susceptibility

	**MICs ≧ 64 mg/l**				**MICs ≦ 8 mg/l**				**Total**	
			
**Source**	***C. albicans***	***C. glabrata***	***C. tropicalis***	**Subtotal**	***C. albicans***	***C. glabrata***	***C. tropicalis***	**Subtotal**	**number**	**%**
Urine	0	5	11	16	4	11	15	30	46	52.3
Sputum	4	1	5	10	5	3	3	11	21	23.9
Blood	0	0	3	3	0	3	0	3	6	6.8
Wound	2	0	0	2	1	0	2	3	5	5.7
Fluid	0	1	0	1	1	0	1	2	3	3.4
Genital tract	1	1	1	3	1	1	1	3	6	6.8
Catheter tip	0	0	0	0	0	0	1	1	1	1.1
**Total**	**7**	**8**	**20**	**35**	**12**	**18**	**23**	**53**	**88**	**100**

MIC, minimum inhibitory concentration; Fluid consisted of ascites, dialysate, and peritoneal fluid.

The analysis of fluconazole susceptibility according to characteristics of different patients is shown in Table 3. There was no significant difference correlated to host factors such as different demographic characteristics and underlying diseases other than the possibility of pulmonary tuberculosis. The observation that patients with pulmonary diseases, particularly those receiving antituberculous agents were found to be more frequently colonized or infected with fluconazole resistant yeasts requires further investigation. Although patients' ages did not significantly differ between two groups, we still adjust its effect in the multivariate analysis because age might influence hosts' underlying conditions the acquisition of infections. In a logistic regression, which included the presence of pulmonary diseases (yes/no), use of antituberculous agents (yes/no) and age (continuous in years), the former two showed some associations but age did not (p = 0.23, details no shown). Use of antituberculous agents was associated with an odds ratio (OR) = 4.2, p = 0.05 for the resistance to the fluconazole, while the presence of pulmonary diseases was with OR = 2.7, p = 0.1.

**Table 3:** Characteristics of patients colonized or infected with *Candida *species

			**Fluconazole susceptibility**				
**Characteristics**	**Total**		**MICs ≧ 64 mg/l**		**MICs ≦ 8 mg/l**		
			
Mean of age (years)	65.5		65.0		66.3		
Duration of hospital stay (days)	58.0 (N = 78)		60.0 (N = 30)		56.7 (N = 48)		
Duration of fever (days)	10.2 (N = 62)		10.7 (N = 24)		9.8 (N = 38)		
	
	**number**	**%**	**number**	**%**	**number**	**%**	**p value**
	
Hospitalization	80	91	32	91	48	90.6	0.81
Medical units	59	75	23	71.2	36	75	0.99
Surgery units	21	25	9	28.8	12	25	0.94
Intensive care units	39	43.3	17	48.6	22	41.5	0.51
Fever (≧ 38°C)	58	72.5	22	62.9	36	67.9	0.79
Age > 65	57	64.8	22	62.9	35	66	0.76
Male	43	48.9	19	54.3	24	45.3	0.41
Mobility affected	60	68.2	23	65.7	37	69.8	0.69
Underlying diseases							
Diabetes	38	43.2	14	40	24	45.3	0.62
Pulmonary diseases	15	17.1	9	25.7	6	11.3	0.08
Hypertension	10	11.4	4	11.4	6	11.3	1
Bacterial infection	57	64.8	22	62.9	35	66.0	0.76
Special medications or managements							
Antifungal agents	18	20.5	7	20.0	11	20.8	0.93
Antibiotics	76	86.4	29	82.9	47	88.7	0.53
Antituberculosis agents	10	11.4	7	20	3	5.7	**0.05**
Dialysis	4	4.6	1	2.9	3	5.7	0.65
Catheter insertion	70	79.6	28	80.0	42	69.3	0.85
Mortality	38	43.2	16	45.7	22	41.5	0.87
**Total**	**88**	**100**	**35**	**100**	**53**	**100**	

## Discussion

It is well established that mechanically supported or immunocompromised patients with invasive devices are at increasing risk of colonization and infection with pathogenic yeasts [5,17]. The findings of this study further suggest that there is no difference in frequency between fluconazole resistant and susceptible *Candida *species to colonize or infect individual patients according to the host factors analyzed. Hence, fluconazole resistant strains do not appear to have more advantage than susceptible ones in these patients. Many nosocomial Candida infections are endogenously acquired [18-21]. It is much more likely that the increase of fluconazole resistant yeasts has resulted from the intense use of fluconazole in response to increasing prevalence of nosocomial yeast infections.

In the previous study, the resistant rate was found to associate with regions and hospital types [13]. In this study, we set out to investigate whether the hosts per se was an influencing factor, since regional difference may be a reflection of host characters. As it turned out, patients receiving antituberculous agents had higher occurrence to be colonized or infected with fluconazole resistant strains. These 10 ten patients receiving antituberculous agents were not associated with either regions or types of hospital.

Among HIV-infected patients, treatment with antituberculous drugs, previous history of tuberculosis, and fluconazole exposure are the risks for development of oropharyngeal colonization or infections by fluconazole-resistant Candida strains [22]. Noteworthily, findings from this study indicate that non-HIV infected patients receiving antituberculous agents had higher occurrence to be colonized or infected with fluconazole resistant strains. Interestingly, a link between mycobacterial infections and oropharyngeal candidiasis in HIV-infected patients has been reported. The prevalence of *Candida *species colonization or infections in AIDS patients in the tuberculous group was 2.5-fold higher than that without tuberculosis [23]. One possible explanation for this phenomenon is that antituberculous agents may change the microbial ecosystem in the body to favor *Candida *species [22]. Antibiotics, such as quinolones, reduces the effects of antifungal agents in a murine model of invasive candidiasis [24]. Hence, antituberculous agents may also antagonize the effects of antifungal agents to prolong survival of *Candida *species and consequently the development of drug resistance. The mechanism by which the usage of antituberculous agents is a risk for colonizing or infecting with fluconazole resistant strains is worthy of further study by a larger series.

## Abbreviations

TSARY: Taiwan Surveillance of Antimicrobial Resistance of Yeasts; MIC: minimum inhibitory concentration; NHRI: National Health Research Institutes; CLSI: Clinical and Laboratory Standards Institute; IRB: Institutional Review Board.

## Competing interests

The authors declare that they have no competing interests.

## Authors' contributions

YLY conceived the study and designed it together with MFC and HJL. MFC, YWC, TGY, HC, SCL, BMHC, TCC, YHH, ZYS, CHHC, and JYL reviewed medical charts of patients. FCT performed the statistical study. YLY drafted the manuscript with contribution from HJL.
